# Anticancer effect of Indanone-based thiazolyl hydrazone derivative on p53 mutant colorectal cancer cell lines: An *in vitro* and *in vivo* study

**DOI:** 10.3389/fonc.2022.949868

**Published:** 2022-08-04

**Authors:** Silpa Narayanan, Qiu-Xu Teng, Zhuo-Xun Wu, Urooj Nazim, Nishant Karadkhelkar, Nikita Acharekar, Sabesan Yoganathan, Najia Mansoor, Feng-Feng Ping, Zhe-Sheng Chen

**Affiliations:** ^1^ Department of Pharmaceutical Sciences, College of Pharmacy and Health Sciences, St. John’s University, Queens, NY, United States; ^2^ Department of Pharmaceutical Chemistry, University of Karachi, Karachi, Pakistan; ^3^ Department of Reproductive Medicine, Wuxi People’s Hospital Affiliated to Nanjing Medical University, Wu-xi, China

**Keywords:** ITH-6, indanone, NF-κB p65, anticancer, tumor xenograft model

## Abstract

Colorectal cancer is a major health problem, and it is the third most diagnosed cancer in the United States. The current treatment for colorectal cancer includes irinotecan, a topoisomerase I inhibitor, and other targeted drugs, such as bevacizumab and regorafenib. The low response rates and incidence of high toxicity caused by these drugs instigated an evaluation of the anticancer efficacy of a series of 13 thiazolyl hydrazone derivatives of 1-indanone, and four compounds among them show favorable anticancer activity against some of the tested colorectal cancer cell lines with IC_50_ values ranging from 0.41 ± 0.19 to 6.85 ± 1.44 μM. It is noteworthy that one of the indanone-based thiazolyl hydrazone (ITH) derivatives, *N*-Indan-1-ylidene-*N’*-(4-Biphenyl-4-yl-thiazol-2-yl)-hydrazine (ITH-6), has a better cytotoxicity profile against p53 mutant colorectal cancer cells HT-29, COLO 205, and KM 12 than a p53 wild-type colorectal cancer cell line, such as HCT 116. Mechanistic studies show that ITH-6 arrests these three cancer cell lines in the G2/M phase and induces apoptosis. It also causes a rise in the reactive oxygen species level with a remarkable decrease in the glutathione (GSH) level. Moreover, ITH-6 inhibits the expression of NF-κB p65 and Bcl-2, which proves its cytotoxic action. In addition, ITH-6 significantly decreased tumor size, growth rate, and tumor volume in mice bearing HT-29 and KM 12 tumor xenografts. Moreover, CRISPR/Cas9 was applied to establish an NF-κB p65 gene knockout HT-29 cell line model to validate the target of ITH-6. Overall, the results suggest that ITH-6 could be a potential anticancer drug candidate for p53 mutant colorectal cancers.

## Graphical Abstract

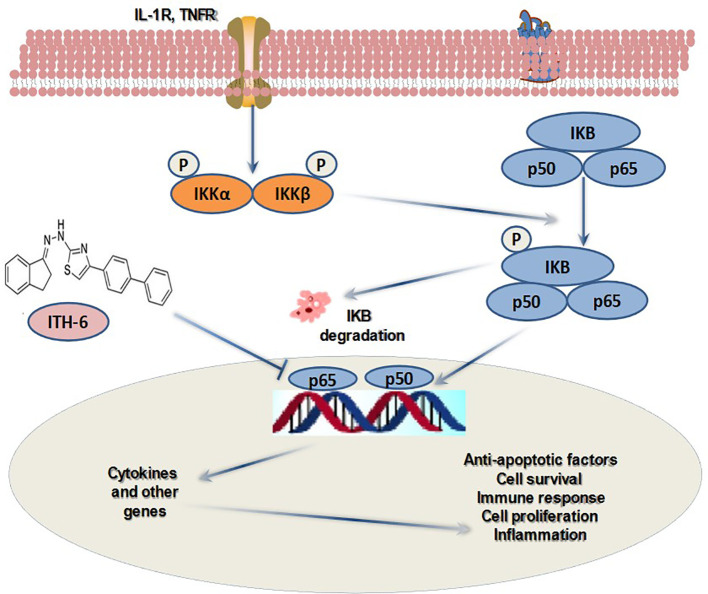

## Introduction

Cancer is the leading disease of human populations regarding the advancement of treatment strategies (1–3). Despite progress in the cancer research field that discovered possible treatments for various cancer types, cancer remains the second leading cause of death after cardiovascular diseases (4–6). In the United States, among malignancies, colorectal cancer (CRC) is the third most common type of cancer, and it consists of a heterogeneous group of tumors, some with gene mutations. According to the data provided by American Cancer Society, there were around 104,610 new CRC cases diagnosed in 2020, and around 53,200 deaths were reported (7). Studies prove that a positive family history increases the risk of occurrence of CRC by approximately 15%–20% (8). The current strategies for the management of primary CRC is the use of a combination of 5-fluorouracil, leucovorin, and either oxaliplatin (FOLFOX protocol) or irinotecan (FOLFIRI protocol) (9–11). These agents exhibit adverse effects, such as vomiting, diarrhea, and other complications, causing a major drawback of the treatment (12, 13). It is reported that the indanone ring exhibits anticancer activity (14–17), and some indanone-related compounds have some crucial bioactivity. Various methods have also been adapted for the synthesis of indanone derivatives as it is a useful moiety. Among the signaling pathways associated with tumorigenesis and inflammation, nuclear factor-kappa B (NF-κB) is a key regulator (18), and the NF-κB family consists of five subunits, RelA (p65), RelB, NF-κB1 (p50 and its precursor p105), NF-κB2 (p52 and its precursor p100), and c-Rel (19). It is established that there is a positive relationship between activation of NF-κB in the intestinal epithelial cells and tumor formation (19), which plays an important role in the occurrence of CRC. NF-κB signaling is associated with a number of responses, including cellular immunity, inflammation, cell differentiation, proliferation, and apoptosis (20–24). This impact on cell proliferation by NF-κB depends on p53 (tumor suppressor gene) status. This is one of the many aspects of the crucial relationship between NF-κB and p53. As studied earlier, wild-type p53 expression opposes NF-κB function and inhibits tumorigenesis, and around half of human cancers exhibit p53 mutations (or have lost the wild-type allele) and, thus, activate the NF-κB pathway during the development of tumors. Moreover, the NF-κB pathway enhances the transcription of mouse double minute 2 (Mdm2), which is a ubiquitin E3 ligase enzyme of p53 and, thus, indirectly helps in regulating the stability of p53 (25). It is established that ITH-6, one of the most active indanone derivatives, arrested the cells at the G2/M phase of the cell cycle and thereby inhibited the proliferation of CRC cells and induced apoptosis by building reactive oxygen species and decreased the intracellular glutathione (GSH) level (26). In the current study, we explore the further mechanism of its anticancer activity by downregulating NF-κB p65 and Bcl-2 expression in *in vitro* and preclinical studies. The current work recognizes the relative part of the NF-κB pathway in cancer and its activation and its effect on downstream target genes, which is crucial for the design and discovery of novel targeted anticancer agents.

## Materials and methods

### Chemicals and equipment

The thiazolyl hydrazone derivatives of 1-indanone were synthesized at the University of Karachi, Pakistan (27). Irinotecan hydrochloride was procured from Alfa Aesar (Haverhill, MA). Dulbecco’s modified Eagle’s medium (DMEM, IX), fetal bovine serum (FBS), phosphate buffered saline (PBS), and trypsin 0.25% were acquired from Hyclone (Waltham, MA). Monoclonal antibodies D97JR (selective against ALDH1A1), E7K2Y (against CD44), D14E12 (against NF-κB p65), E4Z1Q (against topoisomerase I), D3R6Y (against procaspase-3), 44D4 (against IkBα), 16H1 (against GAPDH), D5C9H (against TBP), and secondary anti-rabbit/mouse HRP linked antibody were bought from Cell Signaling (Danvers, MA). A-11005 (alexafluor 594 secondary antibody against NF-κB) and DAPI (catalog # D3571), which stains the nucleus, were purchased from Invitrogen (Waltham, MA). The NF-κB p65, Bcl-2, and 18 S TaqMan gene expression and superscript IV reverse transcription kits were purchased from Fisher Scientific (Waltham, MA).

### Cell lines and cell culture

The human CRC parental cell lines SW620 and S1, ABCB1-overexpressing drug-resistant subline, SW620/AD300 and ABCG2-overexpressing drug-resistant cell line, S1-M1-80 were employed for the ABCB1 and ABCG2 reversal studies, respectively. SW620/AD300 cells were maintained in complete medium with 300 ng/ml of doxorubicin (28). S1-M1-80 cells were expanded in the DMEM medium with the anticancer drug mitoxantrone, starting with a low concentration, and the maximum concentration was 80 µg/ml to induce the ABCG2 transporter expression. These cell lines were obtained from Dr. Susan E. Bates (Columbia University, New York). The cell lines were cultured in DMEM medium containing 10% FBS and 1% penicillin/streptomycin at a temperature of 37°C, 5% CO_2_.

### Experimental animals

Male athymic NCR (nu/nu) nude mice (age 5–7 weeks and weight around 20–25 g) were acquired from Taconic Farms (Albany, NY) for the animal study and remained in polycarbonate cages (four mice/cage) at St. John’s University Animal Care Center. They were maintained under light/dark cycles, supplied with food and water, and monitored for tumor growth by measuring the size using Vernier calipers. The protocol was accepted by the St. John’s University’s Institutional Animal Care & Use Committee (IACUC), protocol #1940. The study was accomplished following the ARRIVE guidelines and Animal Welfare Act and the Guide for the Care and Use of Laboratory Animals.

### Cytotoxicity of ITH-6 on ABCB1 and ABCG2 overexpressing cell lines

The cytotoxicity assay was completed using parental (SW620 and S1) and drug-resistant (SW620/AD300 and S1-M1-80) cell lines that were seeded (6 × 10^3^ cells/well) followed by ITH-6 incubation (with a range of 0–100 µM). After 68 h, the absorbance was detected at 570 nm by spectrophotometer as previously described (29, 30) and IC_50_ values were calculated.

### Western blot analysis

The Western blot assay was conducted to observe aldehyde dehydrogenase 1 family member A1 (ALDH1A1) expression, a cell surface adhesion receptor protein (CD44), a subunit of nuclear factor kappa light chain enhancer of activated B cells (NF-κB p65) (nuclear and cytoplasmic), procaspase-3, topoisomerase I (TOP 1), and IkBα (nuclear and cytoplasmic) proteins after incubating HT-29, COLO 205, and KM 12 cells with three concentrations of ITH-6, 0.3, 1, and 3 µM for 72 hours by a method (31) and further quantified.

### mRNA expression

HT-29, COLO 205, and KM 12 cancer cells were incubated with 0.3, 1, and 3 µM of ITH-6 for 72 hours, and total RNA was extracted using the RNA extraction trizol reagent as previously described (32). The target genes were NF-κB p65 and Bcl-2, and 18S was used as the loading control. The results are represented as relative fold of mRNA expression.

### Immunofluorescence

For this experiment, the cells were cultured with ITH-6 (0.3, 1, and 3 µM) for 72 hours and immunofluorescence performed according to the protocol detailed before (33).

### Molecular modeling

A Macintosh operating system (OS Sierra) with Mac Pro 6-core Intel Xenon E5 processor system was used to perform docking experiments using the Maestro v12. 3. 012 software (Schrödinger, LLC, New York, NY, USA, 2019) software. Lig-prep was used for ITH-6 ligand preparation (34). The heterodimer protein model was imported from the Protein data bank. “Protein Preparation Wizard” was used for protein preparation. Grid generation was done by selecting residues at 20 Å distance from bound inhibitors in the model protein (1IKN) (35). The residues selected were 26, 28, 29, 30, 49, 50, 181, 222, 224, 225, 236, 237, 238, 239, 241, 258, 259, 260, 261, 275. Extra precision docking was performed with a maximum 10 poses.

### Knockout of NF-κB p65 gene in HT-29 cells

A CRISPR/Cas9 system was used to construct the NF-κB p65 gene knockout subline of HT-29 cells. The custom-designed mammalian CRISPR vector was obtained from Vector Builder Inc. (Chicago, IL). The transfection of the NF-κB p65 targeting vector into HT-29 cells was conducted using Fugene 6 transfection reagent (Promega, Madison, WI) according to the manufacturer’s instructions. The knockout of the NF-κB p65 gene was further verified by measuring protein expression using Western blotting and by a cell viability study using MTT.

### Nude mouse MDR xenograft model

The CRC cells, HT-29, and KM 12 xenograft mouse models were established as previously reported (36, 37). The HT-29 (6 × 10^6)^ and KM 12 cells (7 × 10^6^) were implanted into the mice (right and left sides, respectively), and when the tumors attained a diameter of around 0.5 cm (day 0) after one week, the animals were divided into four groups of six each as follows: (a) polyethylene glycol 300 as the vehicle, given orally (q3d × 7); (b) irinotecan (30 mg/kg, q3d × 7), given intraperitoneally (i.p.), dissolved in normal saline (38); (c) ITH-6 (3 mg/kg) dissolved in PEG 300 and given orally (q3d × 7); and (d) ITH-6 (6 mg/kg) dissolved in PEG 300 and given orally (q3d × 7). The treatment period was 21 days. To determine the drug dosage, the body weights were noted every third day. Tumor volumes (using the two diameters of tumors, termed A and B) were recorded every third day using Vernier calipers using the following formula, V = π/6(A + B/2)^3^ (39, 40). Blood was drawn *via* submandibular puncture on the last treatment day using isoflurane inhalational anesthesia, and white blood cell (WBC) and platelet counts were recorded in all groups.

At the end of the treatment period, the animals were euthanized, and the tumors were removed and weighed.

### Collection of plasma and tumor tissues

Mice bearing HT-29 and KM 12 tumors were grouped into three categories: (i) mice receiving 3 mg/kg ITH-6 orally, (ii) 6 mg/kg ITH-6 orally, and (iii) 30 mg/kg i.p. irinotecan. Mice were anesthetized with isoflurane (3%), and 60 µL of blood was taken into heparinized tubes by submandibular puncture at various time points, 5, 30, 60, 120,180, and 240 minutes after the treatment. Moreover, the tumors were removed, weighed, and stored at -80°C for further experiments. The samples were analyzed using high-performance liquid chromatography (HPLC).

Method for Irinotecan: Flow rate: 0.5 ml/min.

**Table:** 

Time (min)	Solvent A percentage	Solvent B percentage
0	60	40
10	98	2
12	98	2
15	60	40

Method for ITH-6: Flow rate: 0.5 ml/min.

**Table:** 

Time (min)	Solvent A percentage	Solvent B percentage
0	60	40
20	98	2
22	98	2
25	60	40

The t_R_ (retention time) for irinotecan was 6.2 minutes and t_R_ for ITH-6 was 17.5 minutes.

The standard curve was created based on dosage: Irinotecan (2 mg/ml, 1 mg/ml, 0.5 mg/ml, 0.25 mg/ml, 0.125 mg/ml, and 0.625 mg/ml) and ITH-6 (1 mg/ml, 0.5 mg/ml, 0.25 mg/ml, 0.125 mg/ml, 0.625 mg/ml, and 0.313 mg/ml).

### Statistical analysis

The experiments were performed at least three times, and the variations were analyzed using one-way analysis of variance (ANOVA). The statistical significance was determined at *p* <.05. The *post hoc* analysis was carried out using Tukey’s test. The data were analyzed using GraphPad Prism, version 6.

## Results

### ITH-6 is not susceptible to ABCB1- and ABCG2-mediated drug resistance

**Figure 1:**
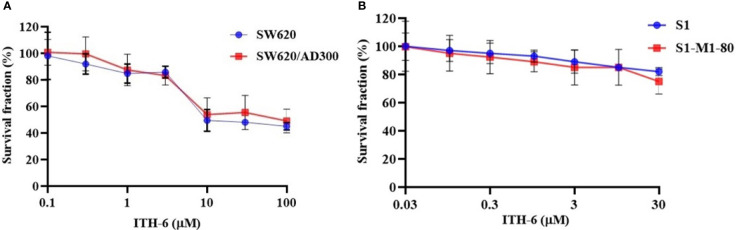
Cytotoxicity of ITH-6 on ABCB1- and ABCG2-overexpressing cell lines. Survival fraction (%) was measured after treatment with ITH-6 (µM) for 72 hours on **(A)** SW620, SW620/AD300 and **(B)** S1, S1-M1-80 cell lines. Points with error bars represent the mean ± SD for independent determinations in triplicate. The figures are representative of three independent experiments.

In order to know if ITH-6 is a substrate of ABC transporters, such as ABCB1 and/or ABCG2, an MTT assay was conducted to determine the susceptibility of ITH-6 to MDR mediated by ABCB1 and ABCG2 transporters. ABCB1 and ABCG2 transporters have established roles in conferring multidrug resistance by lowering intracellular drug accumulation resulting from extrusion of drugs from the tumor cells. Herein, resistance fold (RF) was used to assess if there was any degree of change in the resistance to ITH-6 due to the presence of ABCB1or ABCG2 (41). The results indicate that there was no remarkable difference in the IC_50_ values of ITH-6 in the ABCB1 overexpressing SW620/AD300 cell line (**Figure 1A**) and ABCG2 overexpressing S1-M1-80 cell line (**Figure 1B**) relative to their corresponding parental cell lines, hence proving that it is not a substrate of ABCB1 or ABCG2 transporter.

### The effect of ITH-6 on the expression level of different targets associated with apoptosis of CRC cells

**Figure 2:**
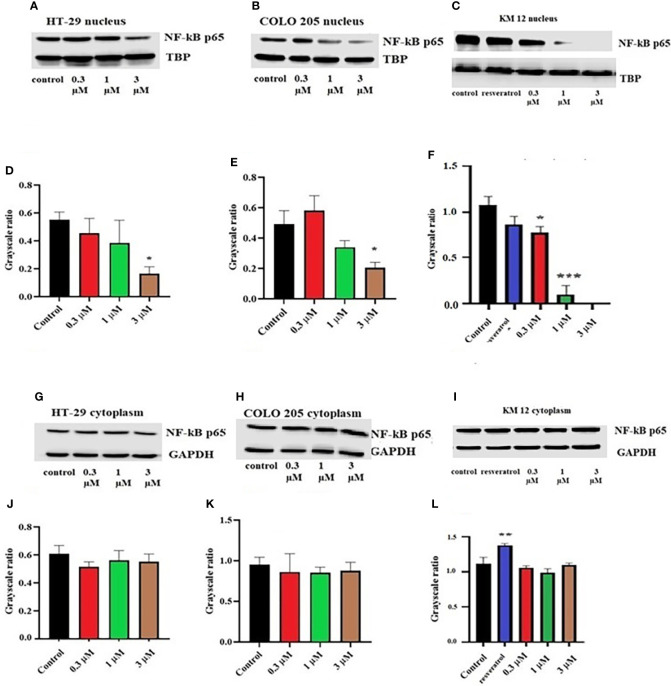
Effect of ITH-6 on the expression of the nuclear fraction of NF-kB p65 protein on **(A)** HT-29, **(B)** COLO 205, and **(C)** KM 12 cells and the cytoplasmic fraction on **(G)** HT-29, **(H)** COLO 205, and **(I)** KM 12 cells. The effect of ITH-6 on the expression of the nuclear and cytoplasmic fraction of NF-kB p65 protein was tested after the cells were treated with 0.3, 1, and 3 mM of ITH-6 for 72 hours. Relative quantification of the effect of ITH-6 on **(D–F)** the nuclear and **(J–L)** cytoplasmic fraction of NF-kB p65 in HT-29, COLO 205, and KM 12 cells. The expression level of NF-kB p65 protein was normalized to TBP (nucleus) and GAPDH (cytoplasm). Equal amounts of total cell lysates were used for each sample, and a Western blot analysis was performed. The figures are representative of three independent experiments. * *p* <.05, ** *p* <.01 and *** *p* <.001 compared with the control group.

To figure out the mechanism of the test drug cytotoxicity, we performed Western blotting on various proteins. The proteins selected were ALDH1A1, CD44, NF-κB p65 (nuclear and cytoplasmic), procaspase-3, TOP 1, and IkBα (nuclear and cytoplasm) as they are important prognostic markers in CRC cell lines. At a concentration of 3 µM, ITH-6 downregulated the nuclear NF-κB p65 expression in HT-29 (**Figure 2A**) and COLO 205 (**Figure 2B**) cells compared with control, whereas in KM 12 cells, the test compound at concentrations of 0.3, 1, and 3 µM significantly decreased the nuclear NF-κB p65 expression level compared with the positive control; resveratrol (20 µM) and KM 12 cells are more sensitive to NF-κB p65 downregulation following treatment with ITH 6 (**Figure 2C**). There was no change in the cytoplasmic NF-κB p65 protein expression in all cell lines treated with ITH-6 (**Figures 2G–I**).

**Figure 3:**
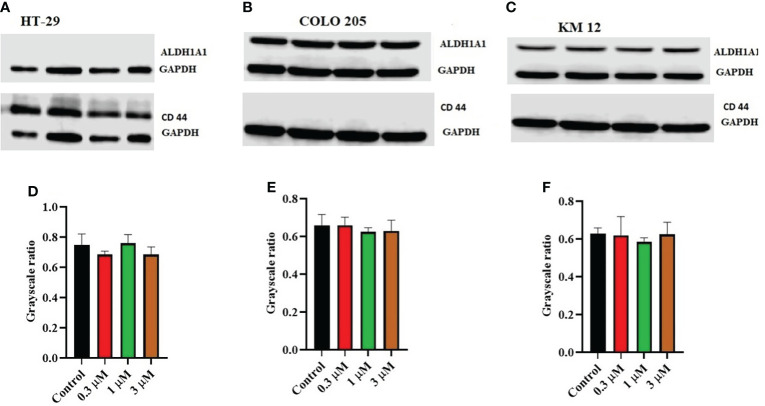
Effect of ITH-6 on the expression of ALD1HA1 and CD44: The effect of ITH-6 on the expression of ALDHA1 and CD44 on **(A)** HT-29 **(B)** COLO 205, and **(C)** KM 12 cells were tested after the cells were treated with 0.3, 1, and 3 μM of ITH-6 for 72 hours. Relative quantification of the effect of ITH-6 on **(D)** CD44 in HT-29 and ALDH1A1 in **(E)** COLO 205 and **(F)** KM 12 cells. The expression levels of the target proteins were normalized to GAPDH. Equal amounts of total cell lysates were used for each sample, and a Western blot analysis was performed. The figures are representative of three independent experiments.

**Figure 4:**
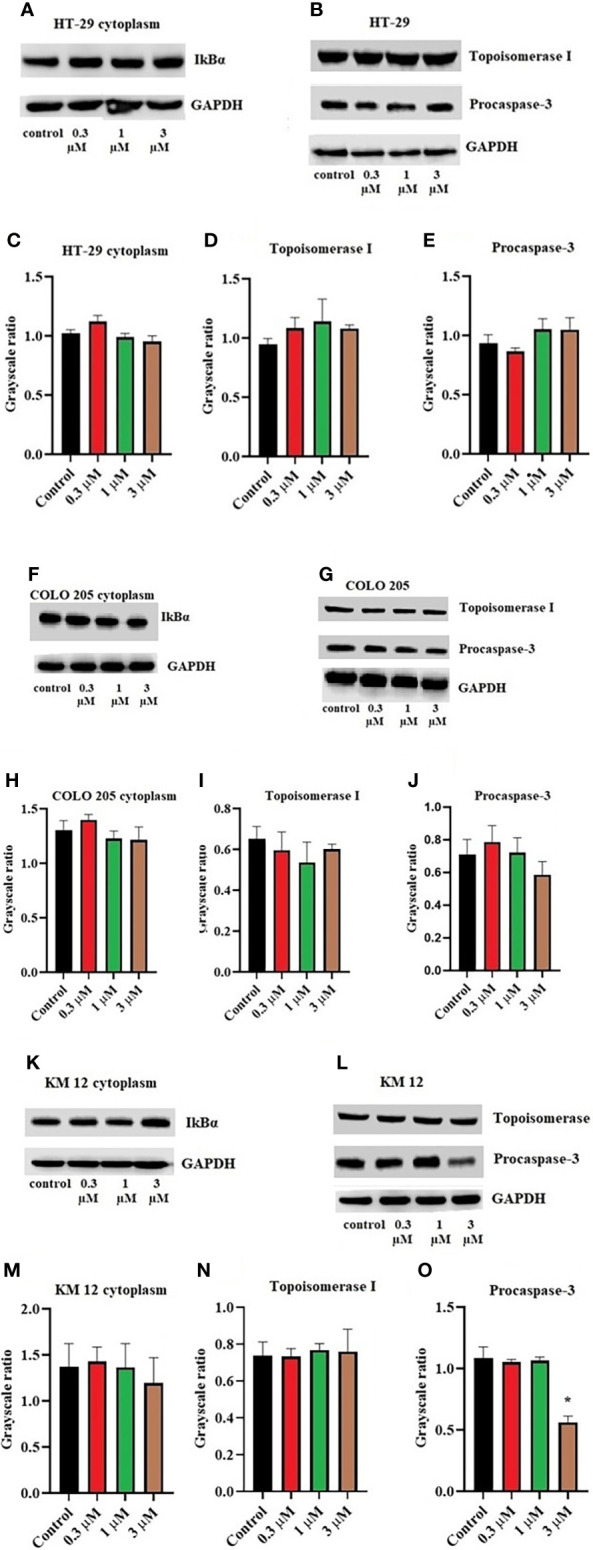
Effect of ITH-6 on the expression of cytoplasmic expression of IkBα, Topoisomerase I, and Procaspase-3 on **(A, B)** HT-29, **(F, G)** COLO 205, and **(K, L)** KM 12 cells. The effect of ITH-6 on the expression of Topoisomerase I, Procaspase-3, and IkBα (cytoplasmic) was tested after the cells were treated with 0.3, 1, and 3 μM of ITH-6 for 72 hours. Relative quantification of the effect of ITH-6 on cytoplasmic IkBα on **(C)** HT-29, **(H)** COLO 205, and **(M)** KM 12 cells, Topoisomerase I on **(D)** HT-29, **(I)** COLO 205, and **(N)** KM 12 cells, and Procaspase-3 on **(E)** HT-29, **(J)** COLO 205, and **(O)** KM 12 cells. The expression levels of the target proteins were normalized to GAPDH. Equal amounts of total cell lysates were used for each sample, and a Western blot analysis was performed. The figures are representative of three independent experiments. * *p* <.05 compared with the control group.

Moreover, there was no change in the expression levels of ALDH1A1 and CD44 (**Figures 3A–C**), TOP 1 (**Figures 4B, G** **, L**), and IkBα (cytoplasmic) levels (**Figures 4A, F** **, K**) on these cell lines. There was a concentration-dependent decrease in the procaspase-3 expression in KM 12 (**Figures 4G** **, L**) cells cultured with ITH-6 at 3 µM for 72 hours, whereas in COLO 205 and HT-29, there was no change in the expression of procaspase-3 after incubating with ITH-6 (**Figures 4B, G**). Hence, we can summarize that the possible mechanism behind ITH-6 induced cytotoxicity in these CRC cells results from downregulating nuclear NF-κB p65 protein expression.

### The effect of ITH-6 on the mRNA level of NF-κB p65 and Bcl-2 in CRC cell lines

**Figure 5:**
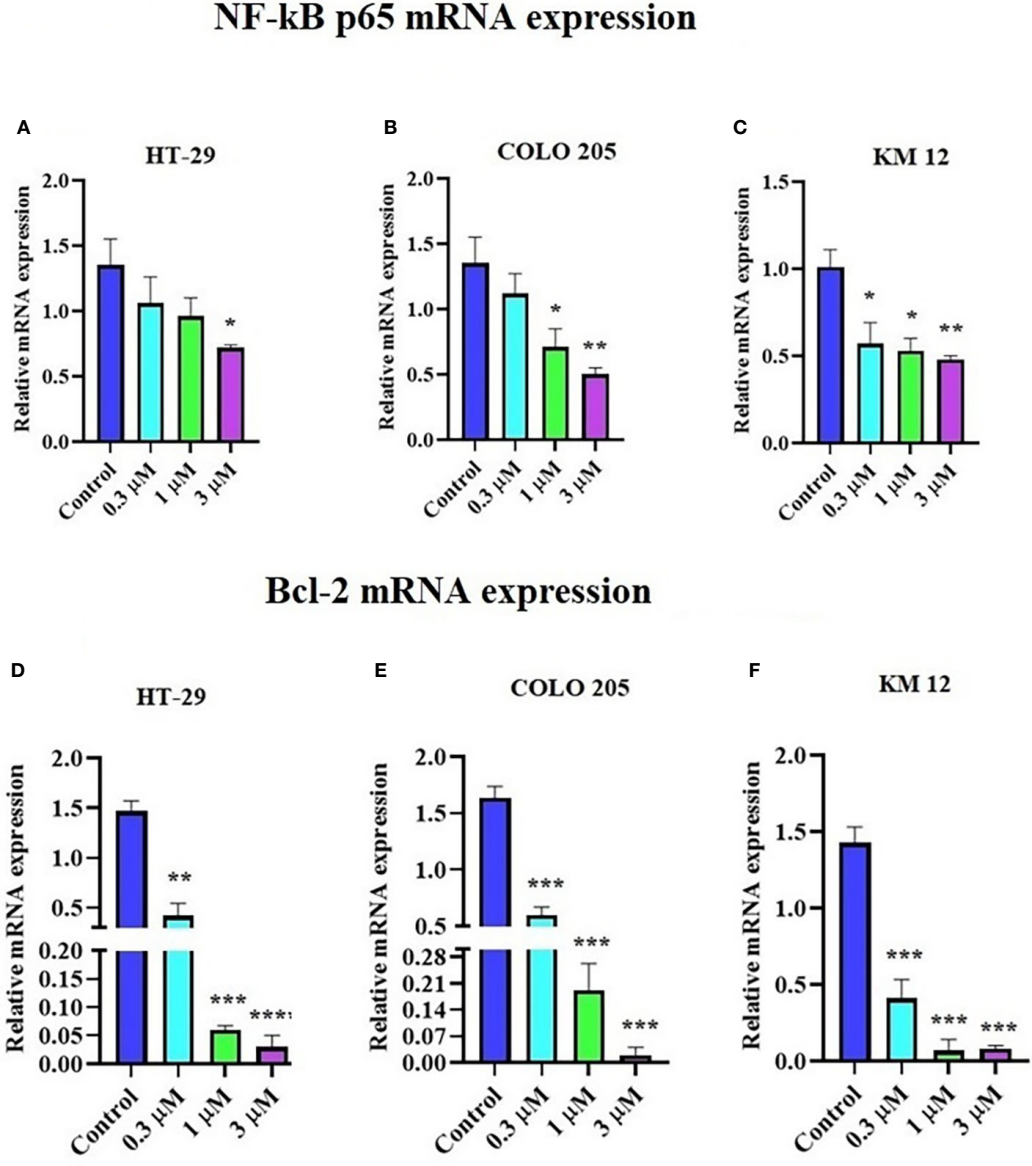
Effect of ITH-6 on NF-κB p65 and Bcl-2 expression at mRNA levels on HT-29, COLO 205, and KM 12 cells. The effect of ITH-6 on NF-κB p65 mRNA expression on **(A)** HT-29, **(B)** COLO 205, **(C)** KM 12 cells, and Bcl-2 mRNA expression on **(D)** HT-29, **(E)** COLO 205, and **(F)** KM 12 cells was tested after the cells were treated with 0.3, 1, and 3 µM of ITH-6 different concentrations for 72 hours. Points with error bars represent the mean ± SD for independent determinations in triplicate. The figures are representative of three independent experiments. * *p* <.05, ** *p* <.01, *** *p* <.001 and **** *p* <.0001 compared with the control group.

The incubation of these three CRC cell lines with 0.3, 1, and 3 µM of ITH-6 for 72 hours remarkably decreased the NF-κB p65 protein expression compared with the vehicle. Furthermore, quantitative real-time PCR (RT-PCR) experiments prove that the treatment of these cell lines with the ITH-6 for 72 hours remarkably decreased NF-κB p65 mRNA expression (**Figures 5A–C**).

It was previously indicated that NF-κB p65 transcriptionally regulates Bcl-2, an anti-apoptotic protein (42). Hence, RT-PCR was performed to evaluate the effect of ITH-6 on the Bcl-2 (**Figures 5D–F**) mRNA level and showed that treatment with ITH-6 downregulated Bcl-2 expression, thereby further proving the role of ITH-6 on the apoptosis of these CRC cell lines.

### Immunofluorescence

**Figure 6:**
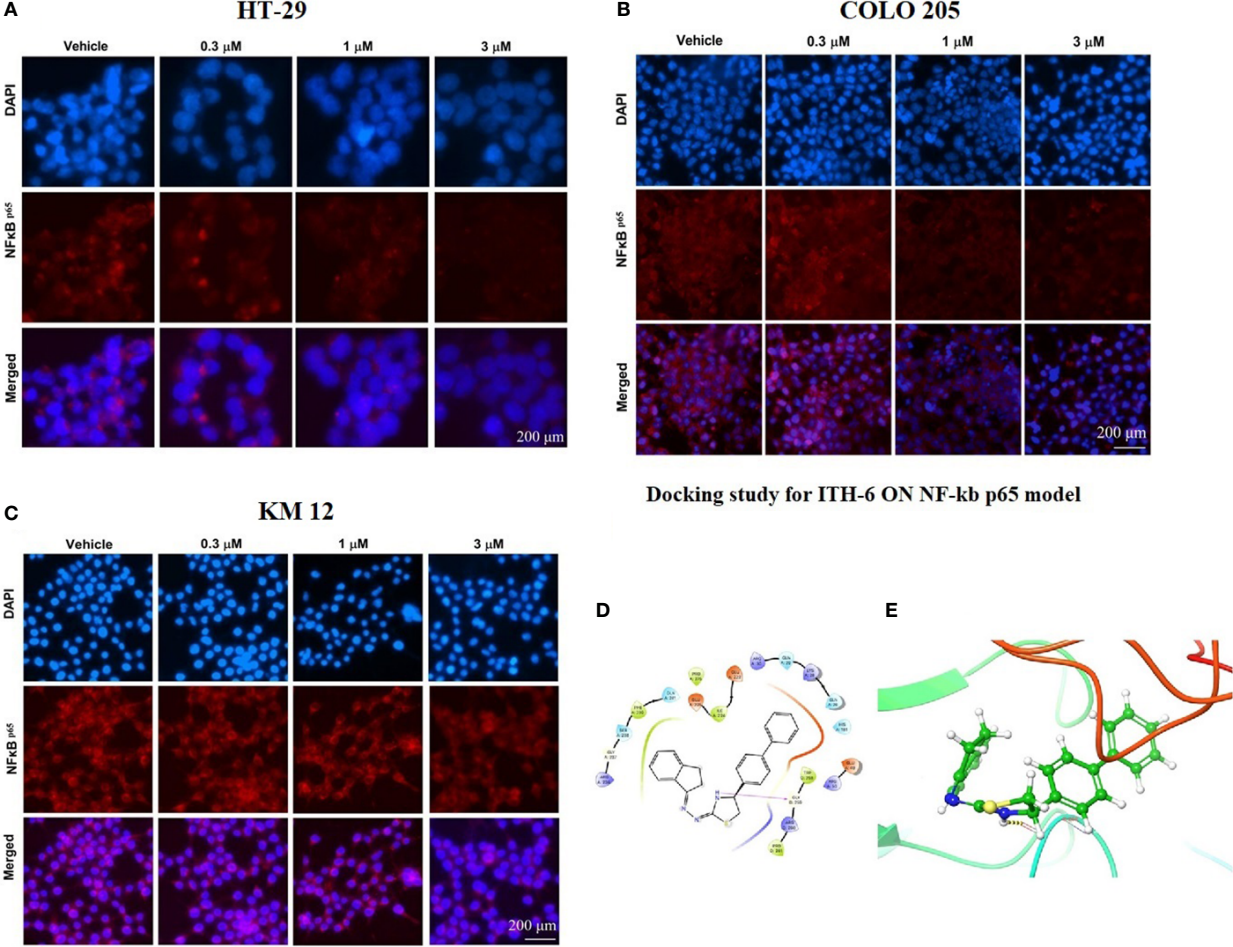
The effect of ITH-6 on the expression of NF-κB p65. **(A)** HT-29, **(B)** COLO 205, and **(C)** KM 12 cells were incubated for 72 hours with 0.3, 1, and 3 µM of ITH-6. The red color represents the presence of NF-κB p65, and the blue color represents the nucleus. **(D)** Docking pose of ITH-6 within the binding pocket of IkBα/NF-κB heterodimer. The protein is represented as multicolored ribbons. Amino acid residues are shown as follows: nitrogen in blue, hydrogen in white, carbon in gray, and oxygen in red. The ligand is represented by the ball and stick model with carbon atoms represented as carbon in green, nitrogen in blue, hydrogen in white, and sulfur in yellow. The yellow dashes represent the hydrogen bonding. **(E)** 2-D ligand interaction between ITH-6 and the IkBα/NF-κB heterodimer. Magenta arrow represents hydrogen bonding with amino acid residues within 5 Å of the ligand.

An immunofluorescence experiment was conducted to find out if ITH-6 can downregulate the expression of nuclear NF-κB p65 in HT-29 (**Figure 6A**), COLO 205 (**Figure 6B**), and KM 12 (**Figure 6C**). Our results confirm that treating these CRC cell lines with ITH-6 decreased NF-κB p65 expression, which is consistent with the Western blot and RT-PCR results.

### Interaction analysis of ITH-6-NF-κB p65 docked complex

The previously reported IkBα/NF-κB crystal model (PDB code: 1IKN) was used for docking analysis. Stimulation between ITH-6 and the heterodimer complex was performed using induced fit docking. The docking position of ITH-6 showed an XP docking score of -5.7 kcal mol^-1^, which shows good binding affinity. **Figure 6D** depicts the docking pose and interaction between ITH-6 and the IkBα/NF-κB heterodimer protein. **Figure 6E** shows H-bonding between the thiazolidine hydrogen and the carbonyl oxygen of GLY259. The biphenyl ring resides in the pocket formed by amino acids: GLN 26, LYS 28, GLN 29, ARG 30, whereas the indene ring sits in the pocket made by amino acids: ARG 236, GLY 237, SER 238, PHE 239, GLN 241.

### Knockout of NF-κB p65 gene in HT-29 cells

**Figure 7:**
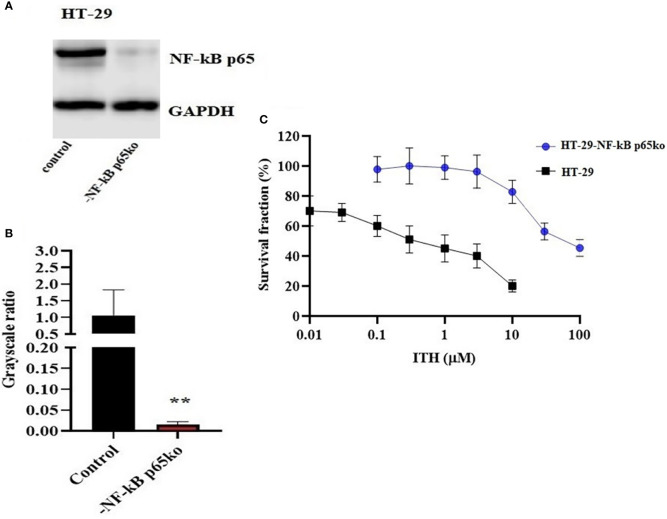
Confirmation of NF-κB p65 knockout in HT-29-NF-κB p65ko cells. **(A)** Western blotting result of NF-κB p65 protein expression level and **(B)** relative quantification of NF-κB p65 in HT-29 and HT-29-NF-κB p65ko cells. The expression level of the target protein was normalized to GAPDH. **(C)** Survival fraction (%) was measured after treatment with ITH-6 (µM) for 72 hours on HT-29 and HT-29-NF-κB p65ko cells. Points with error bars represent the mean ± SD for independent determinations in triplicate. The figures are representative of three independent experiments. ** *p* <.01 compared with the control group.

The knockout of the NF-κB p65 gene in HT-29-NF-κB p65ko cells was verified by the NF-κB p65 protein expression using Western blotting (**Figure 7A**). The level of NF-κB p65 in HT-29-NF-κB p65ko cells was remarkably low compared with that of HT-29 cells (**Figure 7B**).

To further verify the change in gene expression by targeting NF-κB p65 using the CRISPR/Cas9 system in HT-29-NF-κB p65ko cells, MTT assay was performed. RF was used to evaluate if there is any degree of change in the IC_50_ values resulting from the absence of NF-KB p65 expression. Based on the results, the IC_50_ value in HT-29-NF-κB p65ko cells is around 180-fold higher than that of the corresponding HT-29 cell lines (**Figure 7C**).

### The effect of ITH-6 and irinotecan in mice with HT-29 and KM 12 tumor xenografts

**Figure 8:**
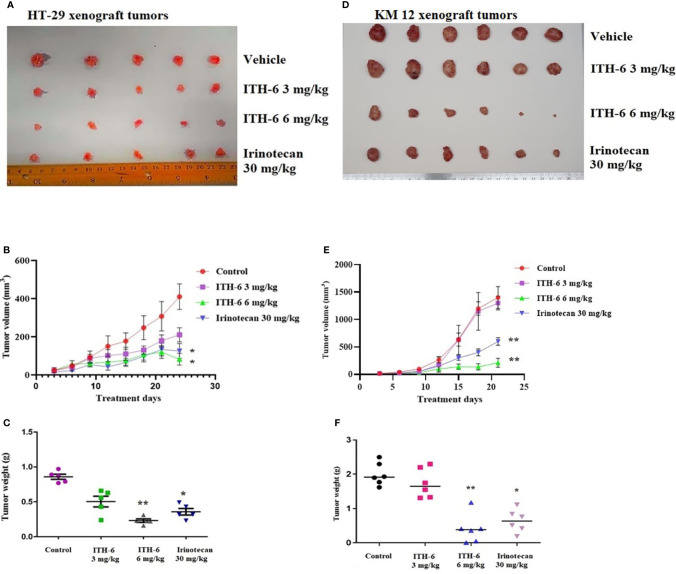
ITH-6 inhibits tumor growth, volume, and weight in xenograft mouse model. NCR nude mice were inoculated with subcutaneous implantation of HT-29 and KM 12 cells. During a 21-day treatment period, ITH-6 (6 mg/kg) significantly inhibited the growth, volume, and weight of **(A–C)** HT-29 and **(D–F)** KM 12 tumor xenografts compared with the vehicle control and irinotecan group. Values represent the median ± SD of six animals per group. Similar results were obtained in two independent experiments. * *p* <.05 and ** *p* <.01 compared with the control group.

**Figure 9:**
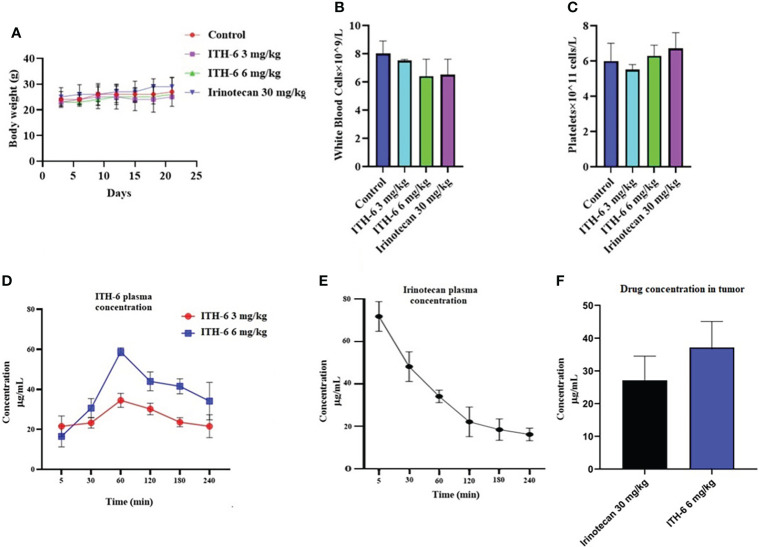
**(A)** Changes in mean body weight before and after treatment for xenograft model are shown. **(B)** The changes in mean white blood cells in nude mice (*n* = 6) at the end of the 21-day treatment period and **(C)** the changes in mean platelets in nude mice (*n* = 6) at the end of the 21-day treatment period. Plasma concentrations of **(D)** ITH-6 and **(E)** irinotecan in nude athymic mice at 5, 30, 60, 120, 180, and 240 minutes following administration of ITH-6 (3 and 6 mg/kg) given orally and irinotecan (30 mg/kg) given intraperitoneally. **(F)** Intratumoral concentrations of irinotecan and ITH-6 in KM 12 (*n* = 6) and HT-29 tumors (*n* = 6) following administration of these drugs. Points with error bars represent the mean ± SD.

We chose irinotecan as a positive control drug because irinotecan is often used for CRC treatment. The two CRC cell lines, HT-29 and KM 12, were implanted, and when the palpable tumors developed, the treatment regimen was started. The mice implanted with HT-29 and KM 12 cells had a significant reduction in volume (**Figures 8B, E**) and weight (**Figures 8C, F**) of the tumor after treatment with an oral dose of ITH-6 6 mg/kg compared with the positive control, 30 mg/kg irinotecan, which was given i.p. (**Figures 8A, D**). The doses that were administered suggest that the drug doses did not produce significant overt toxicity as there was no significant decrease in body weight (**Figure 9A**) and blood cell count (**Figures 9B, C**).

### Concentration of ITH-6 and irinotecan in the tumor and plasma

The plasma level of irinotecan (i.p.) gradually decreased as time increased (**Figure 9E**) and for ITH-6 (given orally), plasma concentration was gradually increasing and reached a peak at 60 minutes and then decreased (**Figure 9D**). However, the tumor concentration of irinotecan (30 mg/kg) was less compared with ITH-6 (6 mg/kg) (**Figure 9F**).

## Discussion

Our previous study found that the drug ITH-**6** has better IC_50_ values on the three CRC cell lines than irinotecan, a clinical-use drug for CRC treatment (26). Indanone-derived compounds have a broader range of different biological activities (43). The IC_50_ values of ITH-6 are 0.44 µM, 0.98 µM, and 0.41 µM on HT-29, COLO 205, and KM 12 cell lines, respectively. Also, the previous data showed the mechanism of ITH-6 on the G2/M cell cycle phase with little effect on other phases and caused an increase in apoptosis.

It is already reported that ITH-**6** induces intracellular ROS production and causes a decrease in GSH levels in all three CRC cell lines with the highest impact at 3 µM (26). Given that the cytotoxicity on CRC cells could result from an inhibition of some specific proteins related to the apoptotic pathway, we conducted Western blotting analysis and RT-PCR to figure out the mechanism of ITH-6. Our results indicate that the incubation of these cancer cells with 3 µM of ITH-6 for 72 hours notably decreased the expression of the nuclear fraction of NF-κB p65 protein compared with cells incubated with vehicle, and the downregulation is more predominant compared with 20 µM of the positive control, resveratrol. There was no significant change in the cytoplasmic level of NF-κB p65 protein (an inactive form that is bound to IkBα). ITH-6 acts only on the nuclear fraction of NF-κB p65, thus proving that it is downregulating the active form of NF-κB p65 protein, which is responsible for the cytotoxicity of ITH-6 on these cell lines.

Moreover, there was no significant change in the levels of ALDH1A1, CD44, IkBα (nuclear and cytoplasmic), TOP I protein upon treatment with ITH-6. The expression of ALDH1A1 was absent in HT-29 cells, whereas CD44 was absent in COLO 205 and KM 12 cells. CD44 could be a surface marker in CRC, and some of the CRC cells could be separated into two populations based on CD44 expression, CD44- and CD44+ (44). ALDH1A1, a member of ALD1H1 family **is** a prognostic predictor of many cancers, including CRC. ALDH1A1 in CRC tissues also has a heterogeneous expression pattern with differences in the rate and intensity. This pattern is also observed in a study of 20 cases of normal colorectal mucosa and 65 cases of CRC and their corresponding adjacent tissues in which ALDH1A1 was upregulated in some cases and downregulated in others (45). There are 14 caspases in mammals. The caspases 8, 9, and 10 are activated by apoptotic stimulation, which activates further effector caspases. Moreover, many studies show that active caspase-3 is needed to induce apoptosis in response to chemotherapeutic treatments. Caspase-3 is synthesized as a 32-kDa proenzyme (procasoase-3), which is cleaved into two subunits and reassociated to form the functionally active caspase-3 enzyme (46). The enzyme TOP1 has a specific role in carcinogenesis. Topoisomerase I (TOP1) cuts one strand in the double-stranded DNA, independent of ATP, and topoisomerase II (TOP2) cuts both strands in DNA and is dependent on ATP for its activity. There are two types of topoisomerases, type I and type II topoisomerase inhibitors interfere with the DNA replication. Topotecan and irinotecan are TOP1 inhibitors and currently used in the treatment of cervical and CRC (47). Inhibitors-of-kappaB (IκB) is an inhibitor of NF-κB and includes various isoforms, such as IκBα, IκBβ, and IκBϵ. IκBα binds to NF-κB in the cytoplasm and blocks the nuclear localization and transcriptional activity of p65. It is only degraded if it is phosphorylated, then ubiquitinated, and finally degraded by the proteasome in a ubiquitin-dependent fashion (48).

The effect of ITH-6 may be on either a transcriptional or translational level. The incubation of the abovementioned cancer cells at various concentrations of ITH-6 for 72 hours decreased the mRNA level of NF-κB p65 significantly compared with cells incubated with vehicle. There was a considerable reduction in the mRNA expression of Bcl-2, which is an anti-apoptotic protein and a downstream molecule of the NF-κB pathway and variety of cancers exhibit a higher expression of Bcl-2 and confer resistance to the apoptotic effect of chemo- and radiotherapy (49).

Subsequently, an *in vitro* immunofluorescent experiment showed that incubation with ITH-6 for a time point of 72 hours decreased NF-κB p65 expression, which is consistent with the Western blot and mRNA expression results. Furthermore, cytotoxicity assays on ABCB1- and ABCG2- overexpressing cell lines showed that there was no significant difference in the IC_50_ values of ITH-6, and it proves that it is not a substrate of ABCB1 or ABCG2 transporter. If a drug is a substrate of ABC transporter, it is more likely to extrude from cells, and that affects the bioavailability of the drug. The results from the gene knockout studies suggest that the NF-κB p65 gene knockout in the HT-29-NF-κB p65ko cell line can be useful in investigating whether ITH-6–induced cytotoxicity is related to the downregulation of the target, NF-KB p65, which is highly expressed in p53 mutant CRCs.

Finally, based on our *in vitro* results, preclinical studies in the athymic nude mice models were conducted to determine the effect of the anticancer effect of ITH-6 on tumor growth on mice implanted with HT-29 and KM 12 cells. The oral administration of 6 mg/kg of ITH-6 reduced the tumor growth remarkably compared with mice that received irinotecan (30 mg/kg i.p.). In addition, no marked change in body weight, WBC, and platelet counts were noted, suggesting that ITH-6 can be well-tolerated at this dose and may be a successful drug candidate for treating p53 mutant CRCs. Furthermore, the anticancer efficacy of ITH-6 is better than the positive control, irinotecan, which can be further proved by its increased tumor concentration compared with irinotecan. These data suggest that ITH-6 has a remarkable anticancer activity in mice with HT-29/KM 12 xenografts at a dose that does not produce notable toxic effects.

Anticancer drug discovery and development are one of the great advancements, and in this manuscript, we demonstrate that ITH-6 is a potent cytotoxic agent against p53 mutant CRC cells and has a preferable cytotoxic profile compared with other drugs that are already approved for CRC. ITH-6 acts on the specific cell cycle phase, causing G2/M phase arrest, and induces apoptosis by elevating the intracellular ROS and decreasing the GSH levels. It also inhibits tubulin polymerization and downregulates the expression of the NF-κB p65 and Bcl-2 in these cell lines, which further proves its role in the cytotoxicity of CRC cell lines. ITH-6 at a dose of 6 mg/kg p.o., did not produce any observable toxic effects in the *in vivo* tumor xenografted mice during the treatment period. It significantly decreased tumor size, growth rate, and tumor volume in mice bearing HT-29 and KM 12 tumor xenografts compared with irinotecan. Together with its mechanism of action, ITH-**6** could be a potential anticancer drug candidate for p53 mutant CRC treatment.

## Data availability statement

The original contributions presented in the study are included in the article/supplementary material. Further inquiries can be directed to the corresponding authors.

## Ethics statement

The animal study was reviewed and approved by St. John’s University’s Institutional Animal Care &amp; Use Committee (IACUC).

## Author contributions

SN and Z-SC: Conceptualization, Methodology, and Experimental design. SN, Q-XT NA and Z-XW: Data Curation. UN and NK: Synthesized the compound, SN and F-FP: analyzed the data and SN, SY,NM and Z-SC: Writing-draft preparation, Reviewing and Editing. All authors read and approved the final manuscript.

## Funding

This project was supported by St. John’s University Research Seed Grant (No. 579-1110-7002), and the Department of Pharmaceutical Sciences at St. John’s University. This study was also funded by the National Natural Science Foundation of China (#81874330) and Wuxi Taihu Lake Talent Plan Top Talents Project (BJ 2020001).

## Acknowledgments

We are grateful to Dr. Susan E. Bates (Columbia University, NY) for the cell lines. We thank Dr. Mansoor Ahmed (University of Karachi, Pakistan) for providing ITH-6 and other synthetic compounds.

## Conflict of interest

The authors declare that the research was conducted in the absence of any commercial or financial relationships that could be construed as a potential conflict of interest.

## Publisher’s note

All claims expressed in this article are solely those of the authors and do not necessarily represent those of their affiliated organizations, or those of the publisher, the editors and the reviewers. Any product that may be evaluated in this article, or claim that may be made by its manufacturer, is not guaranteed or endorsed by the publisher.
